# Estimating brown bear abundance and harvest rate on the southern Alaska Peninsula

**DOI:** 10.1371/journal.pone.0245367

**Published:** 2021-01-29

**Authors:** Earl F. Becker, David W. Crowley

**Affiliations:** 1 Division of Wildlife Conservation, Alaska Department of Fish and Game, Anchorage, AK, United States of America; 2 Division of Wildlife Conservation, Alaska Department of Fish and Game, King Salmon, AK, United States of America; University of South Carolina, UNITED STATES

## Abstract

Abundance estimation of hunted brown bear populations should occur on the same geographic scale as harvest data analyses for estimation of harvest rate. Estimated harvest rates are an important statistic for managing hunted bear populations. In Alaska, harvest data is collected over large geographic units, called Game Management Units (GMUs) and sub-GMUs. These sub GMUs often exceed 10,000 km^2^. In the spring of 2002, we conducted an aerial survey of GMU 9D (12,600 km^2^) and GMU 10 (4,070 km^2^) using distance sampling with mark-resight data. We used a mark-resight distance sampling method with a two-piece normal detection function to estimate brown bear abundance as 1,682.9 (SE = 174.29) and 316.9 (SE = 48.25) for GMU 9D and GMU 10, respectively. We used reported hunter harvest to estimate harvest rates of 4.35% (SE = 0.45%) and 3.06% (SE = 0.47%) for GMU 9D and GMU 10, respectively. Management objective for these units support sustained, high quality hunting opportunity which harvest data indicate are met with an annual harvest rate of approximately 5–6% or less.

## Introduction

Brown bears (*Ursus arctos*) are a long-lived species of low reproductive rate that are managed conservatively in most coastal areas of Alaska, but often without the aid of estimates of population abundance or harvest rate. The Alaska Peninsula, which is Game Management Unit (GMU) 9 managed by the Alaska Department of Fish and Game (ADFG), produces large brown bears that are highly prized by hunters and professional bear guides. To manage this resource more actively for sustained yield and trophy characteristics, accurate and precise estimates of population abundance can be obtained to determine and potentially modify the harvest rates of these populations. ADFG monitors bear harvest on a subunit basis, of which there are 5 in GMU 9 (e.g., GMU 9D). Brown bears are managed for high quality hunting opportunity in GMU 9 and GMU 10 on Unimak Island. Trophy-related management objectives include harvesting a minimum number of large male bears ≥ 8 year of age, males with skull size (length plus width) of ≥ 71.1 cm (28 inches), and a low proportion of females (<40%) in the harvest. The ability to estimate harvest rates allows wildlife managers to determine if changes to hunting regulations are warranted to avoid overharvest. This harvest statistic is not usually available for bear species managed by ADFG. Because of low productivity of brown bears and long recovery periods if overharvested, estimating harvest or exploitation rate can be invaluable for managing this species. During 1998–1999 when annual bear harvest peaked at 353 in GMU 9, Sellers [1] estimated an exploitation rate of approximately 6%; a moderate harvest level which nevertheless continued to maintain trophy-related management objectives. Without estimates of population abundance and harvest rate management is based largely on harvest data indicators (e.g., average skull size and age by sex).

Surveys for brown bears using aircraft are the most cost-effective way to sample large roadless areas that comprise southwest Alaska. Flight paths in mountainous terrain generally follow elevational contours, which allows for safe and efficient sighting of bears in this terrain. In flat terrain, straight line flight paths can be used. Aerial distance sampling has been used to estimate black bear (*Ursus americanus*) abundance in a 23,198 km^2^ area of south-central Alaska [2] and to estimate polar bear *(Ursus maritimus)* abundance in a 465,000 km^2^ area of Southern Hudson Bay, Canada [3]. Distance sampling [4] is a proven method to estimate the size of a population if the assumptions can be met. For conventional distance sampling (CDS) the most important assumption is perfect detection on the transect line [4]. Additional assumptions are random transect location, objects are detected at their initial location, and accurate distance measurements [4].

The ability to achieve perfect detection along transect lines for aerial wildlife surveys is highly doubtful [5]. The biometric literature recommends using Mark-Recapture (MR) methods with mark-resight data [6–8] to assess the perfect detection assumption if any doubt exists as to whether it can be met. If the perfect detection assumption cannot be met, the assumption can be removed by estimating detection on the transect line (apex of detection) with MR methods. These combined distance sampling and MR models are known as Mark-Recapture Distance Sampling models (MRDS; [6,7]) and require covariates in the distance model (MCDS multiple covariate distance sampling) and in the MR model to model detection probability heterogeneity. An MR model that assumes the independence of observations from two observers using the same survey platform over all distances is denoted as the Full-Independence assumption and has been shown to have bias issues due to unmodeled heterogeneity [6,9]. An MR model that only assumes independence of observations among the observers on the line, called Point-Independence, has been shown to avoid many of the issues associated with an MRDS model that assumes Full-Independence [6,10]; for this reason Point-Independence MRDS models are recommended [11]. A critical assumption of MR methods is that, conditional on the covariates in the MRDS model, all objects in the population are equally likely to be “marked” and “recaptured”. Stated another way, after using covariates in the MRDS model to account for differences in detection, no unmodeled heterogeneity exists. If unmodeled heterogeneity exists than negative bias in the estimates of population abundance and precision can occur [11]. For MRDS models using the Point-Independence assumption, this assumption is relaxed to hold just at the apex of detection, usually the transect line [6,10].

Distance sampling detection functions are usually monotonically decreasing; common ones include the half-normal and hazard detection functions [4]. Using fixed wing aircraft with flat side-windows to collect bear distance detection data usually results in unimodal (non-monotonically decreasing) detection shapes [2,12]. Typically, the detection shape is non-symmetrical and unimodal; this type of distance data can be modeled with a gamma detection function [12] or a two-piece normal detection function [2]. When MRDS models with covariates are used, the distance associated with the detection apex varies among covariate levels when a gamma detection function is used. A gamma detection function with covariates has multiple detection apexes, and as a result, a point independence MRDS model cannot be used with this detection function [2]. A two-piece normal detection function, with or without covariates, has one apex at the distribution mode and can be used in MRDS models that assume point independence [2].

We implemented an aerial distance sampling survey in the spring of 2002 to obtain population estimates of brown bears in GMU 9D and GMU 10 (Unimak Island). Our goals for the survey were to obtain unbiased and precise population abundance and harvest rate estimates for each area. In addition, we wanted to examine if the data from these 2 areas could be combined by treating the 2 areas as separate strata [13] and if so, evaluate any gains in precision.

### Study area

**Fig 1:**
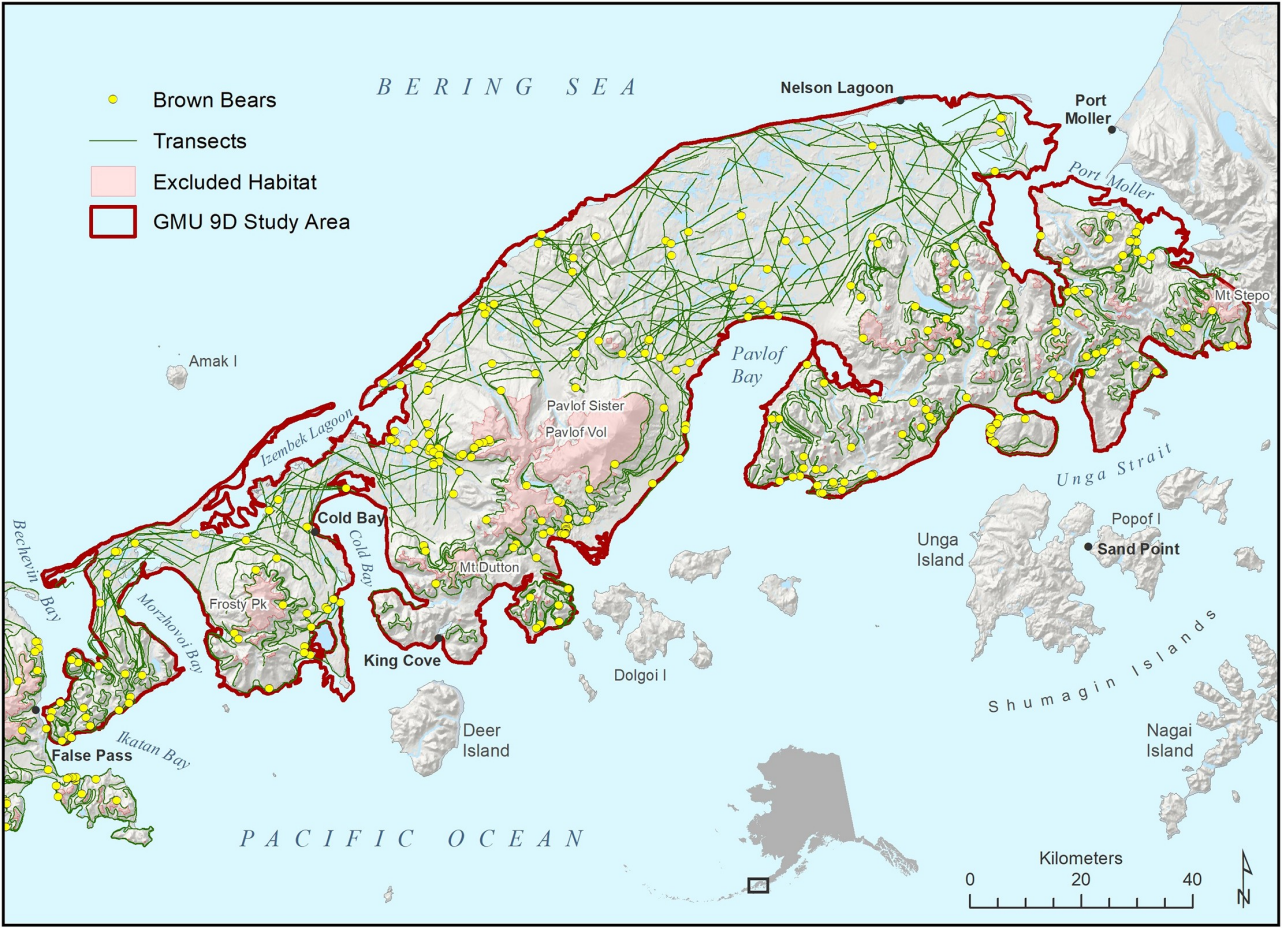
Game Management Unit 9D study area for a spring 2002 distance sampling survey of brown bears, Southern Alaska Peninsula, Alaska (U.S Geological Survey, National Hydrology Dataset https://www.usgs.gov/core-science-systems/ngp/national-hydrography/access-national-hydrography-products and the U.S. Geological Survey, National Elevation Dataset, http://ned.usgs.gov/). Note: Some transects are depicted in very shallow water and low tide mud flats, both of which brown bears use to harvest clams.

**Fig 2:**
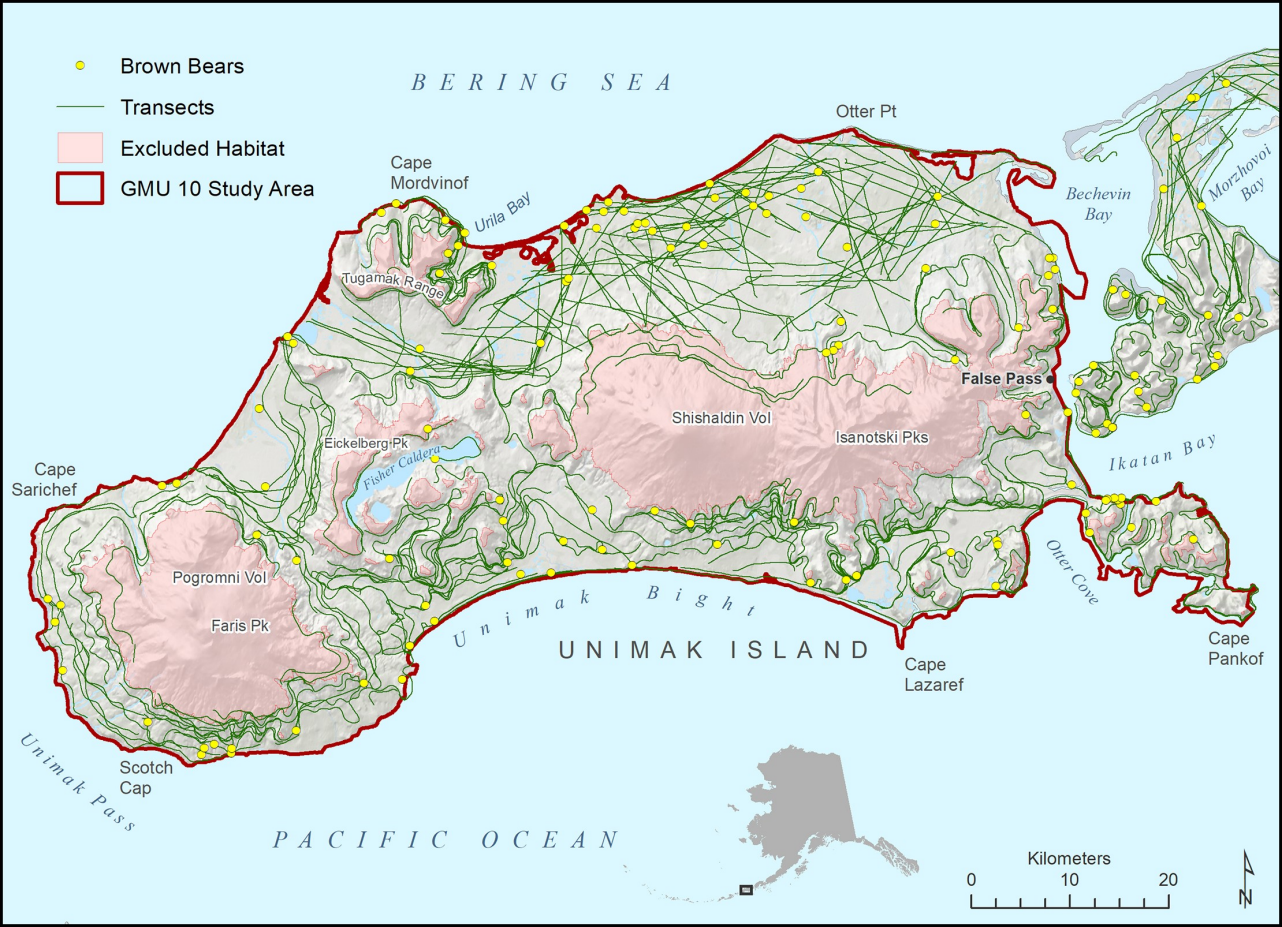
Game Management Unit 10 study area for a spring 2002 distance sampling survey of brown bears, Eastern Aleutian Islands, Alaska (U.S Geological Survey, National Hydrology Dataset https://www.usgs.gov/core-science-systems/ngp/national-hydrography/access-national-hydrography-products and the U.S. Geological Survey, National Elevation Dataset, http://ned.usgs.gov/).

Our study area consisted of 2 areas for which bear abundance estimates were desired. The first was the mainland (non-island) portion of GMU 9D and the second was the portion of GMU 10 which consisted of Unimak Island (Figs 1 and 2). GMU 9D was a 12,599.7 km^2^ area at the end of the Alaska Peninsula. Elevations ranged from 0 to 2518 m; diverse habitats included: cobble and sand beaches, tidal estuaries and lagoons, sand dunes, the Bristol Bay coastal plain of rolling tundra, mountainsides with alternating bare ground of sand, ash and cinder (referred to as cinder blows), alder (*Alnus* spp.), willow (*Salix* spp.) and grass dominated habitats, bare rocky mountain tops and slopes, and snow covered mountains and volcanoes at the highest elevations. Pavlof Volcano on the Pacific side was active and erupts frequently. Three small towns (1,100 people total) with minimal roads are within the GMU 9D study area. Unimak Island was the eastern most Aleutian Island, was 4,069.9 km^2^ large, and the only one inhabited by brown bears. Elevations range from 0 to 2857 m; diverse habitats include: cobble and sand beaches, old lava flows, cinder blows, rolling tundra, mountainsides with alternating alder and grass dominated habitats, bare rocky mountain tops and slopes, and snow-covered volcanoes. Volcanoes are prominent features on the island; Shishaldin Volcano is frequently active. The island is roadless and had about 42 inhabitants in the fishing village of False Pass.

## Methods

We conducted aerial distance sampling, out of Cold Bay, Alaska, in GMU 9D (degrees latitude 52.20, longitude 162.71), from 29 May to 11 June 2002 using 20 km long transects. We used 5-Piper super cub aircraft, a tandem aircraft with a pilot and backseat observer to fly daily surveys. To ensure crew safety, we split the study area up into 5 zones and assigned each plane their own zone to survey the transects located within their zone. If a transect straddled 2 zones, radio-communication between the 2 aircraft ensured it was safely surveyed by one of the 2 planes. Periodically, we shifted zone boundaries to allocate more resources to areas that needed additional resources to complete the survey. No permits were required for this project because surveys were conducted in the air and no animals were pursued or captured. Survey conditions ranged from good to poor; poor conditions included high winds, turbulence, or fog. We instructed crews not to survey transects under poor conditions, instead go to other transects with acceptable conditions and survey them. If conditions for all transects were unacceptable, the aircraft either returned to Cold Bay or landed on the beach to wait for improved conditions. Acceptable conditions included wind and turbulence conditions that allowed the safe and low level (100 m above the ground) flight operation of the aircraft, and sufficient lighting to see bears. The quality of the lighting was variable and included soft warm light, harsher direct sunlight, and flat light. Lighting is hard to quantify; however, crews were instructed to discontinue surveying if they felt lighting conditions were making it too difficult to see bears.

Survey methods to obtain the distance data and mark-resight data and meet MRDS model assumptions was described by Becker and Quang [12]. In the mountainous terrain, contour transects [14] were used. To sample the narrow irregular portions of GMU 9D, straight-line transects were modified to contain a hinge. For each randomly selected midpoint 2 angles were randomly selected to delineate the legs of the transect, if the angle created between the 2 legs was less than 30 degrees, the angle selection process was repeated. If the transect did not fit within the study area, the angle selection process was repeated. Hinged transects in the unexpected mountainous terrain on the western end of GMU 9D, were converted to contour transects by randomly selecting an end point and flying the elevational contour of that point towards the other end of the transect for 20 km. In the study area, brown bear habitat is constrained by elevation, but the upper elevations of bear habitat in these 2 GMUs was unknown. For GMU 10 (latitude 54.82, longitude 164.14), transect selection was restricted to 610 m or lower, and for GMU 9D to 762 m or lower.

Mark-resight data was collected during the distance sampling survey. A screen separated the pilot and backseat observer, and a light system was used to indicate a potential bear sighting. When a potential bear was observed by the pilot or backseat observer, they turned their light on but did not communicate the sighting. When the potential bear was 5 seconds past the trailing wing edge, the sighting was announced, and the lights checked. To get credit for sighting the bear your light must be on. The mark-resight data will be pilot only, backseat observer only or both; essentially each crewmember was marking the bear for the other. After a sighting was announced the plane was flown off transect; if the object was identified as bear(s), a GPS location was collected. The flight path used to get the GPS location was flown parallel to the transect to minimize measurement error on calculating the closest distance to the transect from this location. A location of how far out the crew was searching when the bear was spotted is determined and overflown to get a search distance location. Distance to bear and search distance were calculated using GIS software (ESRI ArcGIS Desktop. Redlands, CA: Environmental Systems Research Institute) as the closest distance from the transect to that location. Additional details are available in Becker and Quang [12].

**Fig 3:**
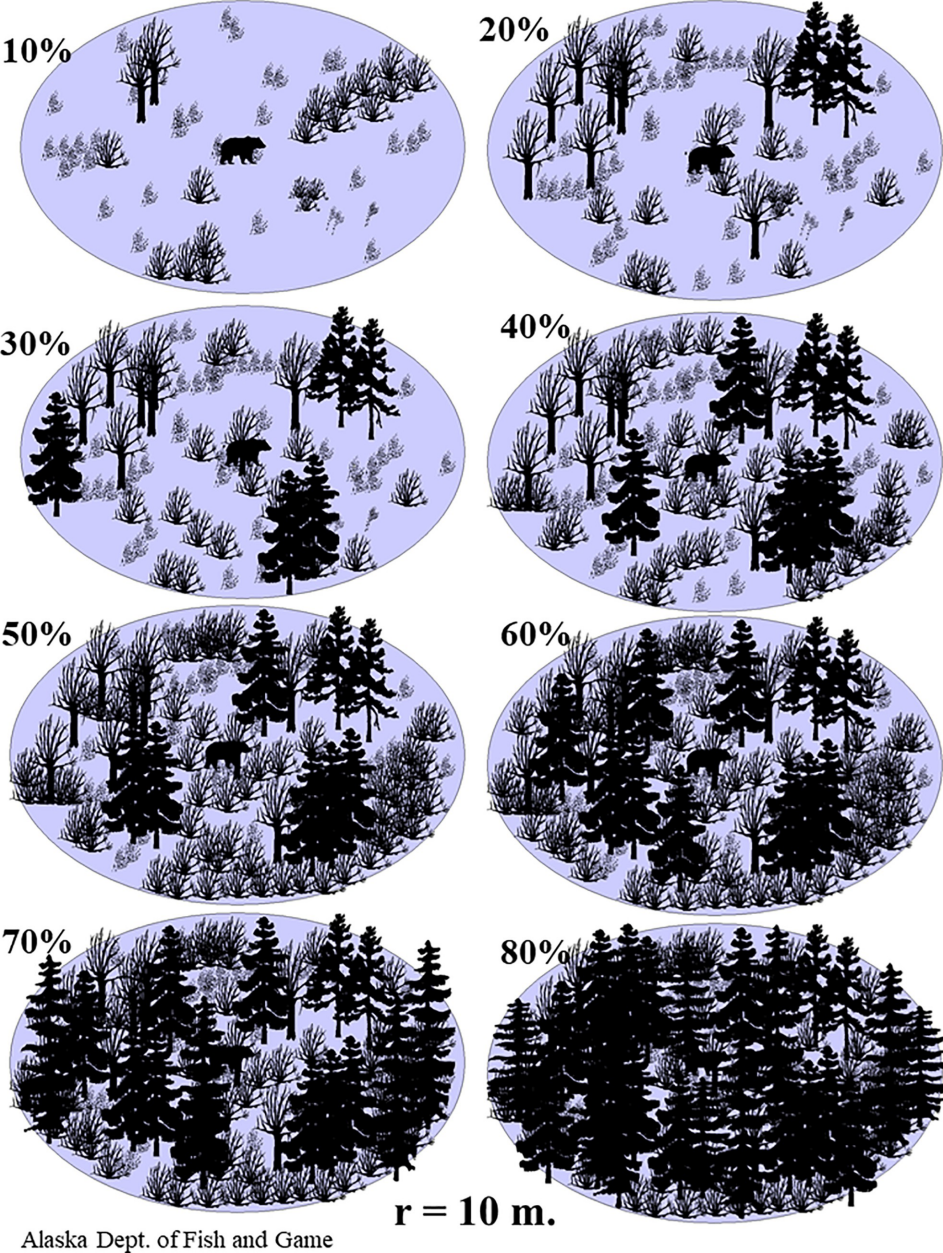
Reference sheet for percent cover and percent snow for a spring 2002 distance sampling survey of brown bears in southwestern Alaska.

Covariates are important for modeling detection probabilities [6,15]. To obtain covariates for each bear group observation, the following data was recorded: bear group size, bear group activity, bear group type, plane speed, percent cover, percent snow, pilot name, backseat observer name, transect type, GMU, and search distance. Bear group activity was recorded as bedded, feeding, standing, walking, or running. Bear group type was recorded as subadult, adult (sex unknown), boar, sow, breeding pair, sow with cubs, sow with yearlings, or sow with 2-year old offspring. Plane speed was the average speed of the aircraft in the 0.5 km transect segment prior to spotting the bear group, it was calculated using the aircraft location and time data. Percent cover included all obscurations and obstructions, with vegetation being the primary cause, within 10 m of the bear group; the crew referred to a reference sheet that depicted percent cover to the nearest 10% (Fig 3). Transect type was recorded as straight or contour. GMU was recorded as 9 for GMU 9D and 10 for Unimak Island.

Some covariates are very difficult to quantify and may even interact with other covariates. Additionally, measuring covariates on the correct scale can be problematic; a bear in 50% vegetative cover within 10 m on an open hillside is easier to detect than one in the same vegetative cover on a hillside with hundreds of patches of 50% vegetative cover. The quality of lighting is hard to quantify but can dramatically affect detection. To account for macro scale and other unmeasured covariates, such as light conditions, we used the search distance covariate. The idea is that if conditions are “good” you can generally search farther than when conditions are average.

Group size was a potential covariate to guard against size-biased sampling [11,16]. Exploratory data techniques, such as stem and leaf plots, LOESS plots, scatter plots, and group summary statistics [17] to identify potentially useful covariates for MCDS and MR modeling. These covariates included modifying the collected covariate data into new covariates. For example, the bear group activity level, bedded bears may have shorter detection distances than other bear activity levels and a new covariate would be created to reflect this. Potential covariates were rescaled to have values which ranged from -5 to 5 to facilitate numerical optimization (J. Laake pers. comm.).

For the MCDS modeling exploratory data analysis identified group size, Pcvr5, PcvrBin3, TranFlat, MCDS2GrpPilot, MCDS2GrpObsvr, Bin2SD1000, and GMU as potential covariates. Pcvr5 was calculated as (percent cover/20). Pcvr5Bin3 was coded 1 for 0% cover, 2 for [10% -30%] cover, and 3 for cover greater than 30%. TransFlat was a covariate which recoded transect type data into a binary variable which was 1 for straight and 0 for contour transects. Exploratory data analysis revealed differences in the distribution of bear detection distances among both pilots and observers. For pilots (n = 5) we created the variable MCDS2GrpPilot which was coded 0 if pilot group = A (n = 1) and 1 if pilot group = B (n = 4). For observers (n = 6) we created MCDS2GrpObsvr which was coded 0 if observer group = A (n = 5) and 1 if observer group = B (n = 1). Exploratory data analysis suggested search distance would be a good potential covariate. In order the met the assumption of independence between covariates and distance for MCDS models [15], we binned search distance into 2 bins and created the covariate Bin2SD1000. We coded Bin2SD1000 as 0 if search distance was 1000 m (*w*) or less, and 1 if it was greater than 1000 m this covariate was denoted as Bin2SD1000. To consider an interaction between IGrMew and Bin2SD1000 we created the covariate ImewBinSD1000 taking the product of the 2 covariates (IGrMew x Bin2SD1000).

For the MR modeling exploratory data analysis identified observer, distance, bed, Pcvr5, MR2GrpPilot, and MR2GrpObsevr. Conditional probability plots by bear activity suggested bedded bears were harder to detect. We created the covariate bed, which was coded 1 for bedded bears and 0 otherwise. Scatterplots of conditional probabilities of detection by pilots and observers suggested differences among each group. For the pilots we created the covariate MR2GrpPilot, which was coded 0 for pilot group a) (n = 1), and 1 for pilot group b) (n = 4). For observers we created the covariate MR2GrpObsevr, which was coded 0 for observer group a) (n = 5) and 1 for observer group b) (n = 1). The covariate observer, coded 0 for pilots and 1 for backseat observers was always used in conjunction with either MR2GrpPilot or MR2GrpObsevr in order to avoid the assumption that pilot group a) and backseat observer group a) had the same conditional probability of detection. Sample sizes of categorical covariates for the MRDS models are listed in Table 1.

**Table 1:** Sample sizes for categorical covariates used in the mark-resight distance models by Game Management Unit, Southwest Alaska.

Covariate	Levels	GMU 9D	GMU 10	Combined
IGrMew	0	37	13	50
	1	216	93	309
BinSD1000	0	218	80	298
	1	35	26	61
TranFlat	0	193	79	272
	1	60	27	87
Bed	0	214	81	295
	1	39	25	64

GMU 9D –denotes Game Management Unit 9D, Alaska Department of Fish and Game, and is at the southern end of the Alaska Peninsula.

GMU 10 –denotes Game Management Unit 10, Unimak Island only, which is the eastern most Aleutian Island and only island in the archipelago inhabited by brown bears.

Natural breakpoints around the 95^th^ percentile of the distance data was used to truncate the dataset and determine the truncation distance, *w* [4]. The two-piece normal detection function was used to fit the MCDS model with parameter *μ* denoting the mode of the distribution and *σ* used as a shape parameter that also incorporated covariates into the model [2]. The covariate IGrMew was used to model the asymmetry of the two-piece normal distribution and was coded 1 if the detection distance exceeded $\hat{\mu }$, 0 otherwise. The effect of search distance may occur only to the right of the detection apex, in which case an interaction between IGrMew and search distance would be required.

A chi-square goodness of fit test [4], a Kolmogorov-Smirnov goodness of fit test, and a q-q plot [18] were used to assess the fit of the MCDS model to the distance data. A chi-square goodness of fit test was used to assess the fit of the MR model to the MR data [6]. Marques and Buckland [19] recommend avoiding models with estimated detection probabilities less than 0.1 or if more than 5% of these probabilities are less than 0.2. On all models with nonsignificant (*α*>0.05) goodness of fit statistics and which had estimated detection probabilities that met the recommendations of Marques and Buckland [19], AIC [20] was used to select the most supported MCDS and MR models for GMU 9D, GMU 10 and the combined data. A forward selection process was used and change AIC < -2 to add an additional covariate [20]. AIC was used to determine if the combined model fit better than the 2 separate models.

Population abundance estimates were obtained using MRDS formulae [6,11] that use a 2-piece-normal detection function [2] and assume point independence [6]. The S1 File was used to perform the analysis. The functions used in the analysis are contained in S2 File, which uses a 2-piece-normal detection function in an MRDS model and allows for stratified sampling to obtain the population estimate and standard errors that include variation in the encounter rate (ER, [number of bears detected/number km transect flown]) [21]. For the combined GMU 9D and 10 analysis, we treated the 2 GMUs as strata to obtain individual GMU estimates. For the separate analyses, the whole dataset is partitioned into 2 subsets (GMU 9D and GMU 10), that was both exhaustive and mutually exclusive of the whole dataset; which allows the AIC statistics for GMU 9D and GMU 10 models to be added and compared to the combined model [20] for both the MCDS and MR models.

Brown bear hunting during the survey period was by biennial registration permit in GMU 9; seasons were open every other regulatory year (RY) (RY01 = 1 July 2001 through 30 June 2002). This GMU 9 restriction for bears, a unique harvest scheme in Alaska, was established nearly 50 years ago to substantially limit harvest. Consequently, annual harvest has been reported as the average for a 2-year hunt period: (closed year + open year)/2. Fall and spring seasons were limited to 2–3 weeks in length. Bear hunting on Unimak Island (GMU 10) was by limited-entry drawing permit with 8 permits available annually. The bag limit for GMU’s 9 and 10 was 1 bear every 4 years. Hunters were required to report their hunting activity including location of kill on a drainage-specific basis. Skulls and hides must be salvaged and presented to ADFG for data collection and tagging with identification. This sealing process was required for transportation of hides and skulls out of state and was therefore a reliable method for monitoring brown bear harvest. Legal (reported) harvest rate was calculated as: (number of legally harvested bears)/(estimated number of bears + number of legally harvested bears). Bear harvest occurred prior to the survey and must be accounted for in the post-hunt population estimate. A 95% harvest rate confidence interval was constructed by dividing the reported harvest by the 95% confidence limits of the population abundance estimate. Harvest rate standard error was calculated by applying the coefficient of variation to the estimated harvest rate. Illegal (unreported harvest) was unknown and not accounted for in our estimates.

## Results

Weather did not allow surveys to be conducted on 1, 9, and 10 June; survey effort was reduced to 3–4 planes on 4, 8, and 11 June due to weather, pilot illness and 2 pilots leaving for other job commitments. Daily local weather within the 5 zones of the study area curtailed survey efforts on an infrequent basis. Not accounting for ferry time to and from the study area, our survey was conducted with approximately 38 plane days of effort. A total of 609 20-km long transects were flown during the survey, with 370 in GMU 9D and 239 in GMU 10. For GMU 9D, no brown bears were detected above 610 m; and for GMU 10 none were detected above 305 m, which we used to define spring brown bear habitat. GMU 9D brown bear habitat consisted of 8,648.2 km^2^ and was sampled with 363 transects with a total length of 7,436.4 km from which 253 bear groups containing 458 bears were detected (Fig 1). GMU 10 brown bear habitat was 2,868.7 km^2^ and was sampled with 219 transects with a total length of 4,393.3 km from which 106 bear groups containing 174 bears were detected (Fig 2).

A stem and leaf plot of the distance data [17] indicated 1000 m was a natural breakpoint in the vicinity of the 95^th^ percentile of the distance data. We used 1000 m for the truncation distance (*w*) and 22 m for the edge of the blind spot underneath the aircraft (*w*_*b*_ = 22,[1]) for the GMU 9D, GMU 10, and the combined dataset; this truncated 5.2% (n = 14) of the GMU 9D data, 5.4% (n = 6) of the GMU 10 data, and 5.3% (n = 20) of the combined GMU 9D and 10 dataset.

**Fig 4:**
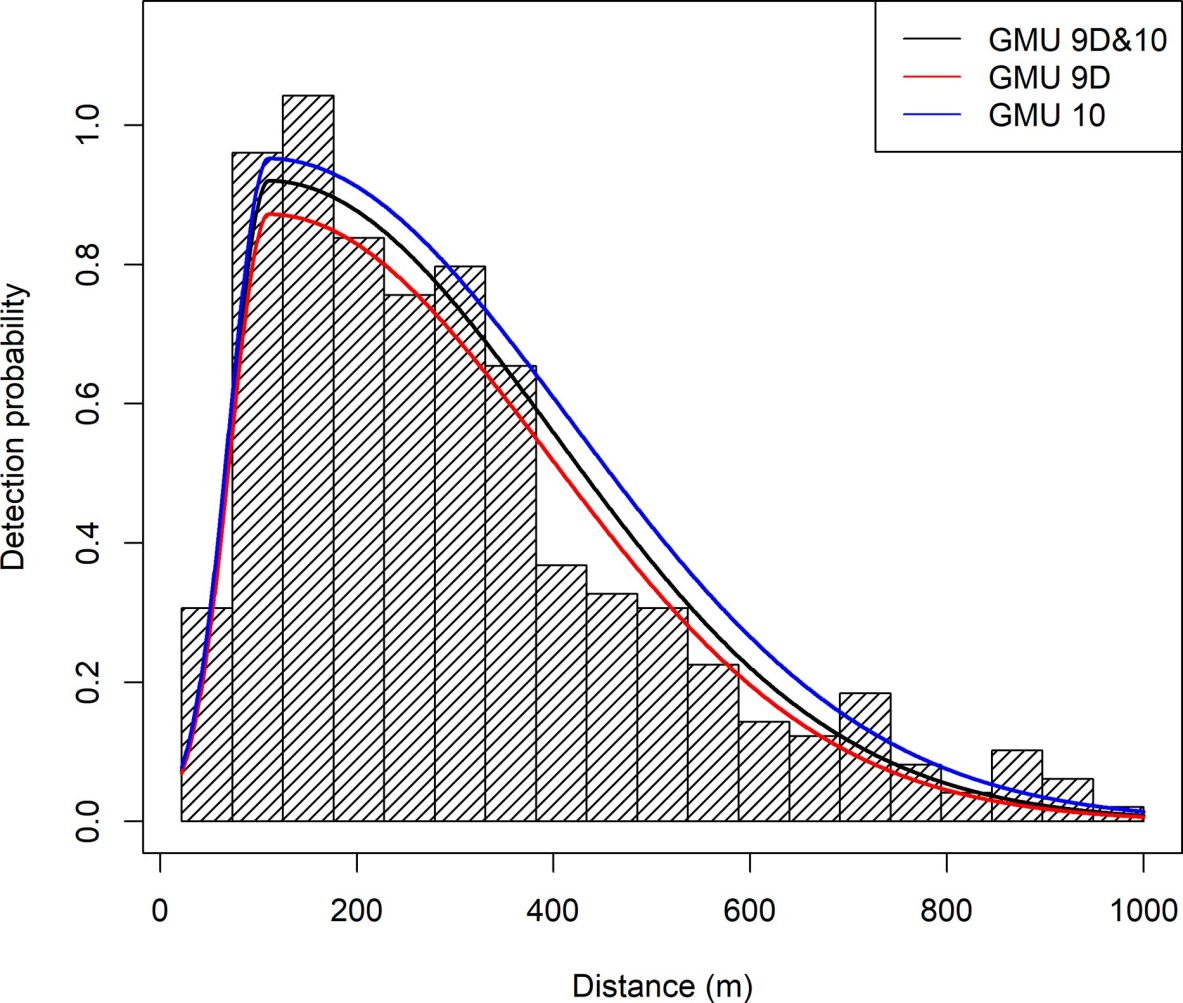
Estimated average detection of brown bears from a spring 2002 line-transect survey of Game Management Unit (GMU) 9D, GMU 10, and for GMUs 9D, and 10 combined, Southwest Alaska. Average detection for the multiple covariate distance sampling model was the mean of the observed covariate values for covariates used in the model applied to their model coefficients. For the mark-resight model, a weighted average of the covariates [2, eq 17) was used to calculate average apex detection, which was used to rescale the graph. The histogram is for the combined GMU 9D and 10 datasets.

Two-hundred and fifty-three bear groups containing 458 bears were detected within the transect searched area (22–1000 m strip next to transect) in GMU 9D, with 204 groups detected by the pilot and 184 detected by the backseat observer, and 135 of these groups were detected by both. For GMU 9D, the most supported MCDS model (AIC = 3262.41) contained an intercept parameter, IGrMew, IMewBin2SD1000, and TranFlat (Table 2). Based on QQ-plots and goodness of fit tests (*χ*^2^ = 10.942, df = 10, p = 0.362; Kolmogorov-Smirnov Statistic p = 0.902) this model fit the data well. The distance of apex of detection ($\hat{\mu }$) was estimated to be 110.6 m. Model fit at the apex of detection is very important [3]. A good fit was obtained with this model (0.860 contribution to the *χ*^2^ statistic of 10.942) for the bin containing the apex. The most supported MR model (AIC = 502.96) contained parameters for the intercept, bed, and Pcvr5 (Table 3). This model fit the MR data well (*χ*^2^ = 23.558, df = 21, p = 0.315) and estimated detection at 110.6 m (${\hat{p}}_{•}\left(\hat{\mu }\right)$) to be 0.872 (SE = 0.024). The combined MRDS model (Fig 4) estimated 1,791.5 brown bears (SE = 189.9), CV = 10.6%, or 207.16 brown bears/1000 km^2^ (SE = 21.96) in GMU 9D.

**Table 2:** Coefficients and standard errors of the best fitting multiple covariate distance sampling (MCDS) models of bear detections in GMU 9D, GMU 10, and GMU 9D and 10 combined southern Alaska Peninsula, Alaska.

Dataset^a^	Parameter^b^	Coefficient	SE
GMU 9D	Intercept	3.6391	0.0940
	IGrMew	1.8268	0.1183
	ImewBinSD1000	0.6395	0.0790
	TranFlat	0.2699	0.0245
	ln($\hat{\mu }$)	4.7062	0.0295
GMU 10	Intercept	3.4151	0.9185
	IGrMew	2.0430	1.0768
	Bin2SD1000	0.6619	0.0769
	TranFlat	0.6218	0.0659
	ln($\hat{\mu }$)	4.7168	0.1397
GMUs 9D & 10	Intercept	3.5823	0.0714
	IGrMew	1.8844	0.0899
	Bin2SD1000	0.6620	0.0412
	TranFlat	0.3724	0.0148
	ln($\hat{\mu }$)	4.7046	0.0215

^a^ GMU 9D denotes Game Management Unit 9D, Alaska Department of Fish and Game, and is at the southern end of the Alaska Peninsula. GMU 10 denotes Game Management Unit 10, Unimak Island only, which is the eastern most Aleutian Island and only island in the archipelago inhabited by brown bears.

^b^ IGrMew: non-symmetrical detection shape parameter coded 0 if distance $<\hat{\mu }$, 1 otherwise, where $\hat{\mu }$ denotes the estimated apex of the detection function (m); ImewBinSD1000: IGrMew x Bin2SD1000, where Bin2SD1000 = 0 if search distance was 1000 m or less, 1 if it was greater; TranFlat: 0 if contour transect, 1 if hinged transect.

**Table 3:** Coefficients and standard errors of the best fitting mark-resight (MR) models of bear detections in GMU 9D, GMU 10, and GMU 9D and 10 combined southern Alaska Peninsula, Alaska.

Dataset^a^	Parameter^b^	Coefficient	SE
GMU 9D	Intercept	1.2052	0.1631
	bed	-1.1579	0.3786
	Pcvr5	-0.4058	0.1622
	${\hat{p}}_{•}\left(\hat{\mu }\right)$	0.872	0.024
GMU 10	Intercept	2.1623	0.4168
	distance	-0.0025	0.0010
	bed	-1.2506	0.4926
	${\hat{p}}_{•}\left(\hat{\mu }\right)$	0.952	0.026
GMUs 9D & 10	Intercept	0.7711	0.7637
	bed	-1.1194	0.2958
	observer	0.2977	0.3776
	MRPilot2Grp	0.8515	0.3905
	distance	-0.0013	0.0006
	Pcvr5	-0.2971	0.1365
	MRObsvr2Grp	1.0011	0.5040
	${\hat{p}}_{•}\left(\hat{\mu }\right)$	0.920	0.019

^a^ GMU 9D denotes Game Management Unit 9D, Alaska Department of Fish and Game, and is at the southern end of the Alaska Peninsula. GMU 10 denotes Game Management Unit 10, Unimak Island only, which is the eastern most Aleutian Island and only island in the archipelago inhabited by brown bears.

^b^ bed: 1 if bear is lying down, 0 otherwise; Pcvr5: percent cover to the nearest 10% divided by 20; distance: closest distance from transect to detected bear group (m); observer: 0 if pilot, 1 if backseat; MR2GrpPilot: 0 if pilot group a, 1 if pilot group b; MR2GrpObsevr: 0 if observer group a, 1 if observer group b; ${\hat{p}}_{•}\left(\hat{\mu }\right)$ denotes the average apex detection (eq 17 in Becker and Christ 2015), where $\hat{\mu }$ denotes the estimated apex of the detection function (m).

One-hundred and six bear groups containing 174 bears were detected within the transect searched area in GMU 10, with 88 groups detected by the pilot and 80 detected by the backseat observer, and 62 of these groups were detected by both. For GMU 10, the most supported MCDS model (AIC = 1388.85) contained an intercept parameter, IGrMew, Bin2SD1000, and TranFlat (Table 2). Based on QQ-plots and goodness of fit tests (*χ*^2^ = 2.340, df = 4, p = 0.673; Kolmogorov-Smirnov Statistic p = 0.275) this model fit the data well. The distance of apex of detection ($\hat{\mu }$) was estimated to be 111.8 m. A good fit at the detection apex was obtained with this model (0.112 contribution to the *χ*^2^ statistic of 2.340) for the bin containing the apex. Our MR data indicated the most supported MR model (AIC = 197.18) contained parameters for an intercept, distance, and bed (Table 3). This model fit the MR data well (*χ*^2^ = 14.518, df = 13, p = 0.338) and estimated ${\hat{p}}_{•}\left(\hat{\mu }\right)$ at 111.8 m to be 0.952 (SE 0.026). The combined MRDS model (Fig 4) estimated 309.9 brown bears (SE = 53.2), CV = 17.2%, or 108.03 brown bears/1000 km^2^ (SE = 18.53) in GMU 10.

**Fig 5:**
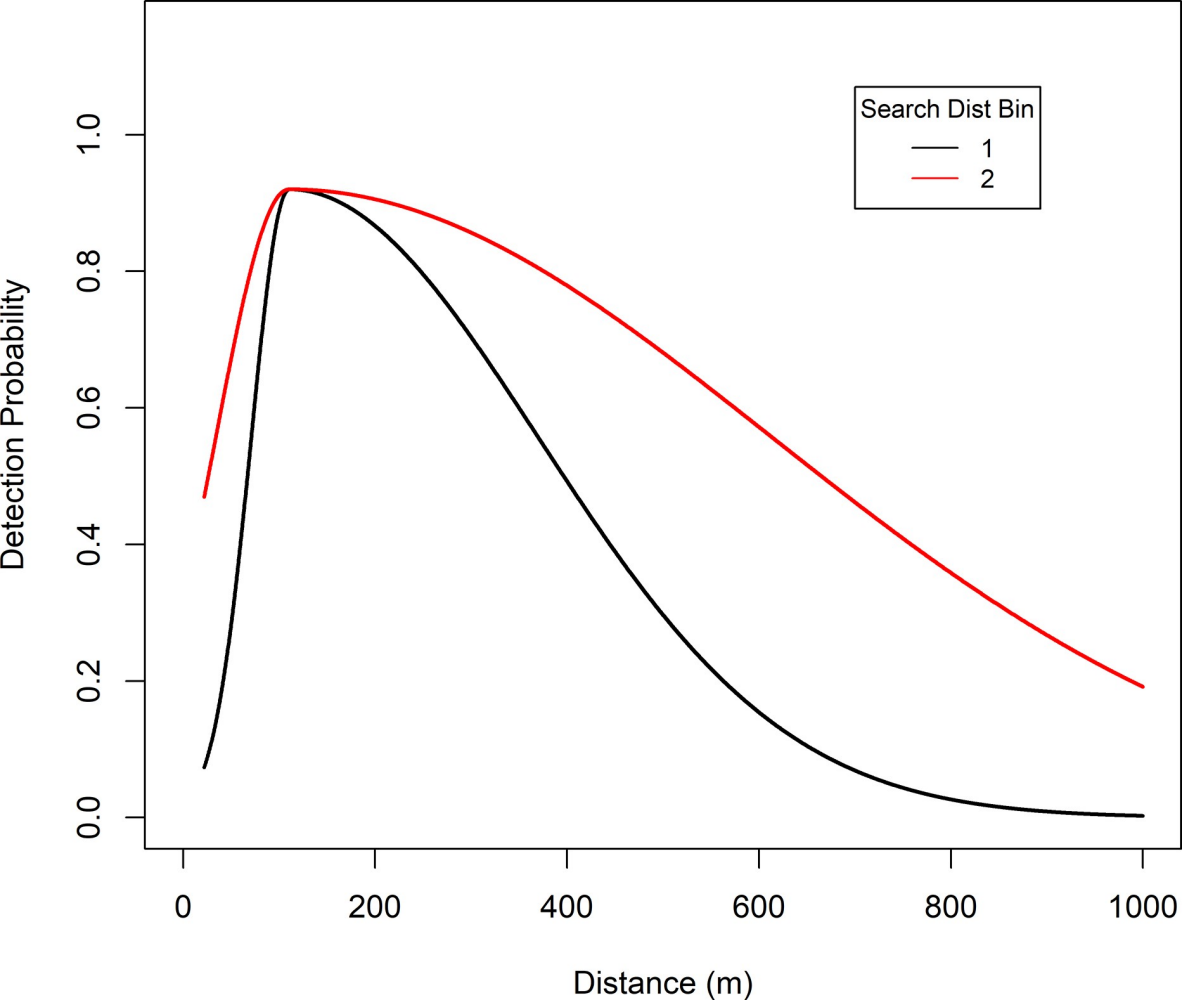
Estimated detection of brown bears in Game Management Unit 9D and 10, Southwest Alaska, by binned search distance, from a spring 2002 distance sampling survey. Bins (Bin2SD1000) were coded as: Bin 1 = 0 (search distance was 1000 m or less), Bin 2 = 1 (search distance greater than 1000 m).

**Fig 6:**
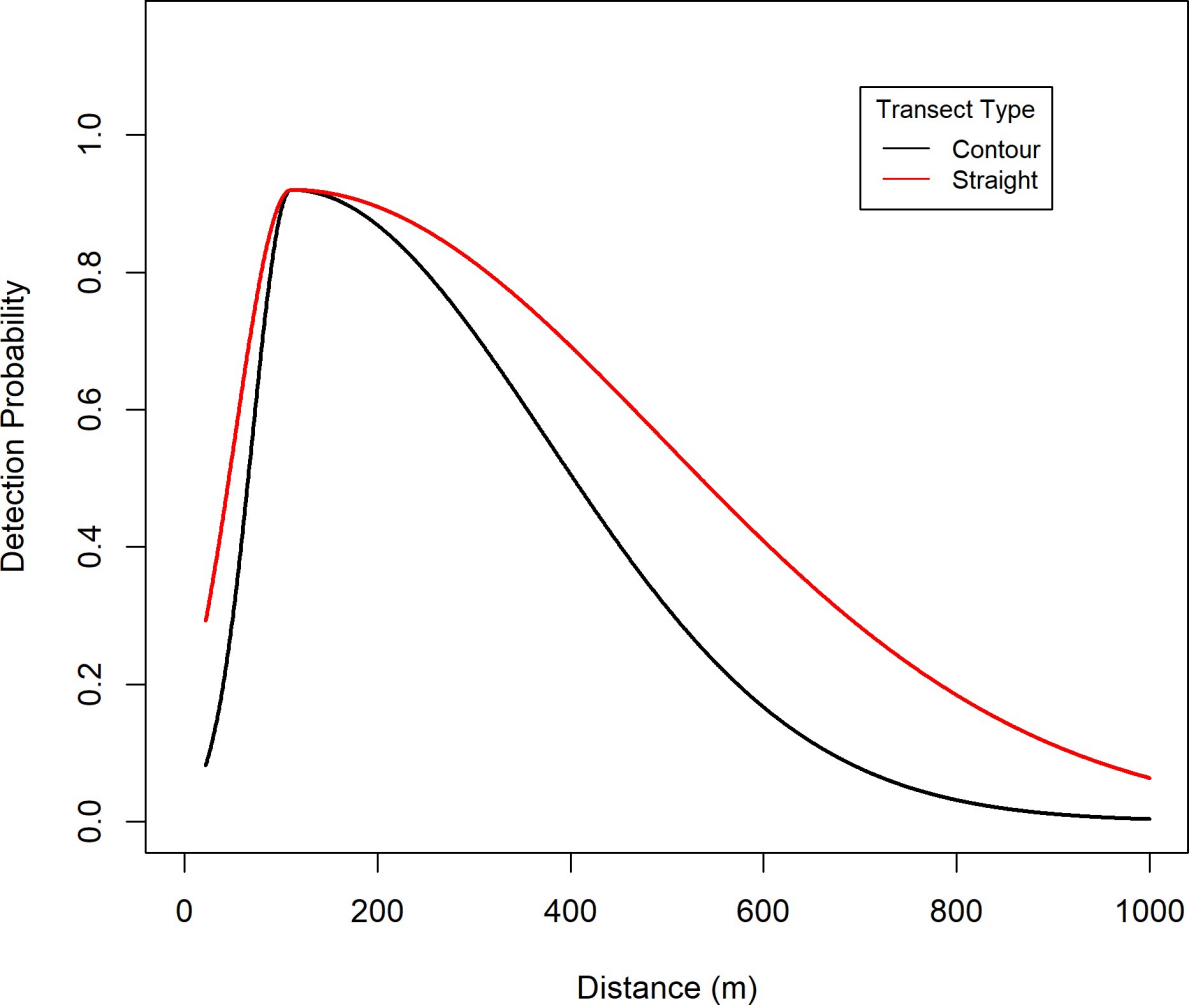
Estimated detection of brown bears in Game Management Unit 9D and 10, Southwest Alaska, by transect type, from a spring 2002 distance sampling survey. Contour transects were flown in mountainous terrain; straight/hinged transects were flown over flatter terrain.

**Fig 7:**
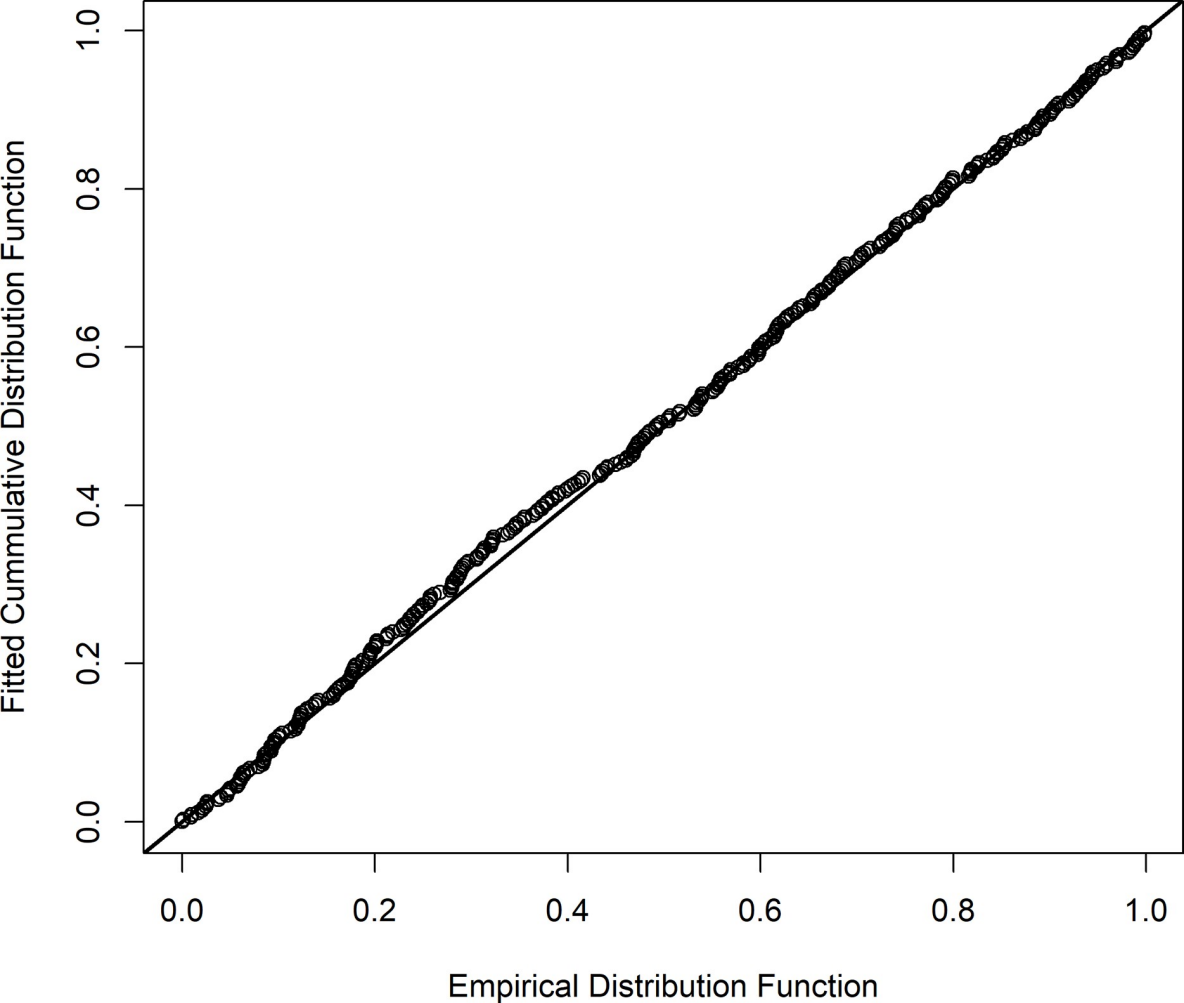
QQ-plot of the best multiple covariate distance sampling model of bear detection in Game Management Unit 9D and 10 combined, Southwest Alaska.

We fit an MRDS model to the combined GMU 9D and GMU 10 dataset. The most supported MCDS model AIC = 4644.75) contained an intercept parameter, IGrMew, Bin2SD1000, and TranFlat (Table 2, Figs 5 and 6). Based on QQ-plots (Fig 7), and goodness of fit tests (*χ*^2^ = 11.566, df = 13, p = 0.563; Kolmogorov-Smirnov Statistic p = 0.645) this model fit the data well. The distance of apex of detection ($\hat{\mu }$) was estimated to be 110.5 m. A good apex fit was obtained with this model (0.437 contribution to the *χ*^2^ statistic of 11.566) for the bin containing the apex. The most supported MR model (AIC = 692.14) contained parameters for an intercept, bed, observer, MRPilotGrp2, distance, Pcvr5, and MR2GrpObsevr (Table 3). This model fit the MR data well (*χ*^2^ = 20.590, df = 21, p = 0.484) and estimated (${\hat{p}}_{•}\left(\hat{\mu }\right)$) at 110.5 m to be 0.920 (SE 0.019). The combined MRDS model estimated 1,682.9 brown bears (SE = 174.3), CV = 10.4%, or 194.60 brown bears/1000 km^2^ (SE = 20.15) in GMU 9D. The combined MRDS model estimated 316.9 brown bears (SE = 48.2), CV = 15.2%, or 110.47 brown bears/1000 km^2^ (SE = 16.82) in GMU 10. The combined MRDS model (Fig 4) estimated 1999.8 brown bears (SE = 186.0), CV = 9.3%, or 173.64 brown bears/1000 km^2^ (SE = 16.15) in GMUs 9D and 10 combined.

Plots of the combined GMU and individual GMU MRDS detection models (Fig 4) indicate the detection models are similar. Comparison of these MCDS AIC values (change AIC = -6.51) indicate the combined GMU 9D & 10 model (AIC = 4644.75) is superior to 2 separate AIC models for GMU 9D and 10 (AIC = 3262.41+1388.85 = 4651.26). Comparison of these MR AIC values (change AIC = -8.00) indicate the combined GMU 9D & 10 model (AIC = 692.14) is superior to 2 separate AIC models for GMU 9D and 10 (AIC = 502.96+197.18 = 700.14). The 95% confidence interval of the combined MR model estimated ${\hat{p}}_{•}\left(\hat{\mu }\right)$ at 110.5 m (0.920, SE 0.019, 95CI: 0.884, 0.957) contained the ${\hat{p}}_{•}\left(\hat{\mu }\right)$ estimates for the GMU 9D (0.872, SE 0.024) and GMU 10 (0.952, SE 0.026) MR models. The combined model was more precise for the individual GMU population estimates (Table 4) than models based on the individual GMU data. The precision gain was slight for GMU 10 (change CV = 0.0192, 1.92%) and miniscule for GMU 9D (change CV = 0.0024, 0.24%). The precision of the combined population estimate (CV = 9.3%) was better than the individual GMU estimates (Table 4) and is due to stratifying on GMU ERs. For GMU 9D, the encounter rate is 0.0616 (SE = 0.0055, CV = 8.9%) bears encountered per km-transect surveyed, and 0.0396 (SE = 0.0054, CV = 13.5%) for GMU 10. Substituting groups for individuals we calculated the group ER as 0.0340 (SE = 0.0028, CV = 8.2%) for GMU 9D and 0.0241 (SE = 0.0029, CV = 12.0%) for GMU 10. No selected MCDS models contained group size as a covariate, so no adjustments were needed to control this potential bias.

**Table 4:** Brown bear abundance estimates, standard errors, coefficients of variation, and confidence intervals for Game Management Unit (GMU) 9D, for GMU 10, and for GMUs 9D and 10 combined, Southwest, Alaska. Line transect survey conducted 29 May to 11 June 2002.

Model Dataset^a^	Estimate	Abundance Estimate	SE	CV	Lower 95% CI	Upper 95% CI
GMU 9D & 10	GMU 9D	1,682.9	174.29	10.36%	1,373.9	2,061.6
GMU 9D & 10	GMU 10	316.9	48.25	15.23%	235.2	427.0
GMU 9D & 10	GMU 9D & 10	1,999.8	185.98	9.30%	1,666.7	2,399.6
GMU 9D	GMU 9D	1,791.5	189.92	10.60%	1,455.6	2,205.0
GMU 10	GMU 10	309.9	53.16	17.16%	221.7	433.3

^a^ GMU 9D denotes Game Management Unit 9D, Alaska Department of Fish and Game, and is at the southern end of the Alaska Peninsula. GMU 10 denotes Game Management Unit 10, Unimak Island only, which is the eastern most Aleutian Island and only island in the archipelago inhabited by brown bears.

Calculating bear harvest rate for GMU 9 is complicated by hunts that are primarily open every other regulatory year as a means to limit hunter harvest. The reported hunter harvest for GMU 9D was 153 bears (69 in fall and 84 in spring) for RY01 and zero in RY02 (Table 5). Average annual harvest for these 2 years was (153 + 0)/2 = 76.5 bears. The resulting harvest rate was 4.35% (se = 0.45%, CV = 10.4%, Table 5) of the estimated population size. The estimated harvest rate in GMU 10 for RY01 was 3.06% (se = 0.47%, CV = 15.2%, Table 5).

**Table 5:** Estimated brown bear harvest rates for Game Management Unit 9D and 10 in Southwest Alaska, October 2001 –May 2003. Game Management Unit 9D has biennial hunts, resulting in open hunts during even years in spring and odd years in fall; therefore, reported harvest over 2-year open and closed periods are averaged to reflect annual harvest. GMU 10, Unimak Island, has annual brown bear hunts.

	GMU 9D^a^	GMU 10^b^
Population Estimate (Spring 2002)	1,682.9	316.9
Coefficient of variation (CV)	0.1036	0.1523
95% Lower Confidence Limit	1,373.9	235.2
95% Upper Confidence Limit	2,061.6	427.0
Reported Harvest (Fall 01-spring 02)	153	10^c^
Reported Harvest (Fall 02-spring 03)	0	10^d^
Average (2-year) Reported Harvest	76.5	10^e^
Estimated Harvest Rate	76.5/(76.5+1682.9) = 4.35%	10/(10+316.9) = 3.06%
se(Estimated Harvest Rate)^f^	0.45%	0.47%
Lower Confidence Limit	76.5/(76.5+2061.6) = 3.58%	10/(10+427.0) = 2.29%
Upper Confidence Limit	76.5/(76.5+1,373.9) = 5.27%	10/(10+235.2) = 4.08%

^a^ GMU 9D denotes Game Management Unit 9D, Alaska Department of Fish and Game, and is at the southern end of the Alaska Peninsula.

^b^ GMU 10 denotes Game Management Unit 10, Unimak Island only, which is the eastern most Aleutian Island and only island in the archipelago inhabited by brown bears.

^c^ Reported harvest used in Estimated Harvest Rate.

^d^ Reported harvest not used in Estimated Harvest Rate.

^e^ Average reported harvest not used in Estimated Harvest Rate.

^f^ Calculated by taking the product of the Population Estimate CV and the Estimated Harvest Rate.

## Discussion

The goal of this survey was to estimate brown bear population abundance on a sub-GMU basis to calculate harvest rates and confidence intervals. To our knowledge, this was the first time in Alaska that brown bear abundance was estimated on the same geographic scale as was harvest data, which allowed for harvest rates and confidence intervals to be calculated. The point estimates for GMU 9D and 10 were below 5% indicating a that harvest level supporting trophy-related objectives occurred in 2002 when bear harvest remained historically high. Despite the age of the abundance estimate (summer 2002) the results of this survey are of interest to present day brown bear management on the Alaska Peninsula. Harvest management has remained conservative; meanwhile the average annual harvest of brown bears in GMU 9D was relatively stable from 2002 to 2013 (range: 126–155 bears) then declined substantially from 118 in RY’s 14 and 15 2015 to 53 in RY 2019, driven primarily by declining number of hunters (D.Crowley unpublished data). In GMU 10 annual harvest was 4–10 bears since 2002; harvest lows for both GMU’s in regulatory year 2019 resulted from the State of Alaska closing its borders to nonresident hunters in spring 2020. Estimating abundance of bears in GMU 9D again using MRDS while harvest is historically low would be of benefit in monitoring the bear population and evaluating this technique.

A secondary goal of this work was to determine if data from the 2 surveys could be combined and if so, the resulting precision gain. The MRDS model with combined data from GMUs 9D and 10 were more supported (AIC) than individual models for each GMU. The combined model for these data produced precise (CV = 10%) results for GMU 9D (CV = 10.4%) and less precise estimates for GMU 10 (CV = 15.2%). Fewster et al. [21] states “the dominant source of variance in distance sampling is usually the encounter rate”. Our results support this observation since the estimated CV of the encounter rates for GMUs 9D and 10 (8.9% and 13.5% respectively) were only slightly smaller than the CV of the population estimate, which incorporates model variance and encounter rate variance. This would explain the small gains in precision we obtained from using a combined model versus single GMU based models; since the estimates of encounter rate and variance are calculated within GMU, and only model variance would differ between the two approaches. This survey had the same truncation distance, crews, and survey dates the survey was conducted differ among surveys which usually results in it being inadvisable to combine these data.

Our MRDS model fit the combined data and had parameter estimates that make biological sense. Our MCDS model indicated that detection probabilities increased when conditions allowed for farther distances to be searched more effectively (Bin2SD1000 = 0.6620, Table 2), similarity straight line transects were more efficient (TranFlat = 0.3724, Table 2) for detection than contour transects in mountainous terrain. Our MR model estimated apex detection at 0.920 (0.019, Table 3) indicating the need to estimate this parameter and how erroneous an assumption of perfect detection would be. The need to estimate apex detection has been documented in other distance sampling surveys of bears [22]. The MR model also indicated bedded bears were harder to detect than more upright bears (bed = -1.1194, Table 3), as distance increased more bears were missed (distance = -0.0013, Table 3), and bears were harder to detect as percent cover increased (Pcvr5 = -0.2971, Table 3). By adding the coefficients for observer, MRPilot2Grp and MRObsvr2Grp (Table 3), we obtain the following cumulative detection coefficients: Pilot Group a (0), Backseat Group a (0.2977), Pilot Group b (0.8515), Backseat Group b (0.2977+1.0011 = 1.2988, Table 3). Pilots have a better view than backseat observers; however, one pilot (Pilot Group a, n = 1) was suffering from the flu during the survey, additionally one backseat observer (Backseat Group b, n = 1) did exceptionally well detecting bears.

When the data was collected, the plan was to obtain a population estimate with an MRDS model based on a gamma shaped detection function [12]. In the process of analyzing the data we were puzzled how a MCDS model could obtain a bigger abundance estimate than one from our MRDS model. Eventually we read and understood Borchers et al. [6] that indicated the full independence assumption of MRDS models was the cause of this bias. It made no sense to publish an analysis which was biased, but we did not have a detection function that could implement the recommended solution of an MRDS model that assumed point independence [6]. A gamma detection function has an apex for every covariate level [2] while the assumption of point independence allows only 1 apex [6]. At this point there was no existing solution on how to model this data. In 2011, the senior author and S. Buckland (University of St. Andrews) collaborated on an effort to use a Level of independence modeling approach [23] based on a logistic detection function for modeling the bear detection data. Initially, progress was made, but ultimately the logistic function was not flexible enough to model the shape of the bear detection data. In desperation, the senior author used a half normal detection function to fit the data, the cost was an additional left truncation of 11.6% to 27.2% of the data which was deemed very inefficient [22]. The left truncated data was also a half normal distribution with a different scale parameter. This insight lead to the development of the two-piece normal detection model [2]. The r package mrds [24] which performs MRDS modeling could not be easily adapted to the two-piece normal detection function; so major r-code writing was required to fit an MRDS model that uses a two-piece normal detection function, graph model results, and perform goodness of fit tests. The complexity of the r-coding coupled with lack of r-coding expertise by the senior author slowed progress in reporting an MRDS model based on a two-piece detection model 2]. The variance in the two-piece normal MRDS model [2] was based on Borchers et al. [6]. The ER variance work of Fewster et al. [21] was not incorporated into the initial MRDS model based on a two-piece normal detection function [2]. The r-code in this paper corrects that error and can analyze stratified data. To summarize, the 2002 data was collected just prior to a time of rapid advancement in MRDS models which caused delays in obtaining a model that was suitable for these data; the complexity of the r-coding caused additional delays in the analysis, this delayed analysis addresses the issues raised above to obtain scientifically sound abundance and harvest rate estimates.

The GMU 9D and GMU 10 survey took 38 plane days to accomplish and detected 359 bear groups that were used in the analysis. Currently survey aircraft with gas charge $375 an hour, assuming 8 hours of flying per day and 38 plane days puts the survey cost at $114,000. The lack of knowledge about brown bear distribution in the study area resulted in a simple random sample (SRS) survey design which is an inefficient design [13].

Survey cost is a major impediment to using distance sampling for brown bear management. The individual GMU 9D and 10 results indicate 253 detections and 106 detections from an inefficient SRS survey resulted in CV’s of 10.60% and 17.20% respectively (Table 4). Survey costs can be managed and reduced by: 1) stratifying on encounter rate; 2) reducing the number of detections for the desired sample size for planning a survey; 3) use better survey designs to more uniformly spread transects out within strata, 4) lowering the desired precision, 5) conducting the survey over 2 or 3 years to make the cost more manageable and treat the abundance estimate as an average [12], and 6) sharing costs with the USFWS when surveying National Wildlife Refuges such as GMU 9D and Unimak Island. Successful stratification will require pre-survey information form a previously done survey or a pilot study [25]. We stratify on encounter rate versus density [26] since encounter rate is responsible for 68% to 90% of the CV of abundance from SRS distance sampling surveys (E.Becker unpublished data). Examination of Fig 1 indicate large geographic areas with lower ERs than other areas. ERs by 250 feet elevational bands ranges from 0.050 to 0.092 for GMU 9D and 0.028 to 0.046 for GMU 10. Clearly ER can be stratified for these areas. It is hard to determine the effect of stratified designs on precision which would impact the setting the desired target sample size, The results of SRS design on the GMU 9D and 10 induvial estimates suggest 200 to 250 detections is a good target range for planning a survey and can be refined once more data is available. Spreading transects out in a more uniform coverage pattern would help with survey precision [25], the use of zig-zag transects [26] in U-shaped river valleys of interior Alaska and constricted flat areas like the narrow portions of GMU 9D (Fig 1) would allow for consistent survey effort in these areas. Precision goals of 10–12% CV seem reasonable.

We should be able to reduce sample size which would reduce precision and gain that precision back with a good stratification plan. Stratifying the study area based on encounter rate by elevational band and sampling proportional to the encounter rate we crudely estimated 65% of our 2002 survey effort would yield about 250 group observations to build a detection model. We hypothesis that the modeling variance would increase slightly, and the encounter rate variance would be significantly decreased. If this conjecture is true, a stratified survey would of GMU 9D and 10 would take 24.7 plane days and cost approximately $74,000; we would anticipate a 10% to 12% CV for GMU 9D and GMU10. Incorporating spatial components into the stratification would improve the efficiency of a new survey and possibly farther reduce costs.

The r-package dssd [25] can implement transect study designs with both straight and zig-zag transects and also depict transect coverage, currently contour transects are not supported. Density surface models [27,28] can be used to increase the precision of the abundance estimates and estimate abundance in sub-regions.

Managing brown bear populations in large remote areas is extremely difficult without reliable abundance estimates. An unbiased and precise estimate of population abundance on the same geographic scale on which harvest data is collected allows for precise estimates of reported harvest rates. The ability to estimate harvest rates is an invaluable tool for wildlife managers to determine if changes to hunting regulations need to be made relative to over or under harvest. Stratified aerial distance sampling with MR data should be considered for these areas. Care must be taken to ensure these surveys can met assumptions. We anticipate costs can be reduced and efficiency increased in future surveys.

## Supporting information

S1 File An r-script file containing the r-code used in the analysis of this manuscript. The r-packages mrds [24] and optimx [29,30] are required to run the analyses.
(R) Click here for additional data file.

S2 File An r-script file containing the functions called by GMU9D10_MRDS_Analysis.r. This code is an improvement of the r-code found in [2]. The most important improvements are the incorporation of encounter rate variance into the population estimate and the ability to use stratified designs. Additional refinements are documented by comments in the -r-code. The r-package mrds [24] is required.
(R) Click here for additional data file.

S3 File A csv file containing the brown bear distance data that was analyzed in this manuscript; this file is required by GMU9D10_MRDS_Analysis.r. (CSV)

S4 File A csv file containing the transect data that was analyzed in this manuscript; this file is required by GMU9D10_MRDS_Analysis.r. (CSV)

## References

[1] Sellers RA. 2003 Unit 9 brown bear management report Pages 103–113 *in* Healy C., editor. Brown bear management report of survey and inventory activities. 1 July 2000–30 June 2002. Alaska Department of Fish and Game, Juneau, Alaska.

[2] Becker EF, and Christ AM. 2015 A unimodal model for double observer distance sampling surveys. PLoS ONE, 10, e0136403 10.1371/journal.pone.0136403 26317984 PMC4552872

[3] Obbard ME, Stapleton S, Middel KR, Thibault I, Brodeur V, and Jutras J. 2015 Estimating the abundance of the Southern Hudson Bay polar bear subpopulation with aerial surveys. Polar Biology 38:1713–1725.

[4] Buckland ST, Anderson DR, Burnham KP, Laake JL, Borchers DL, and Thomas L. 2001 Introduction to distance sampling. New York, Oxford University Press.

[5] Alpizar-Jara R, and Pollock KH. 1996 A combination of line transect and capture-recapture sampling models for multiple observers in aerial surveys. Environmental and Ecological Statistics, 3, 311–327.

[6] Borchers DL, Laake JL, Southwell C, and Paxton CGM. 2006 Accommodating unmodeled heterogeneity in double-observer distance sampling surveys. Biometrics. 62:1207–1220. 10.1111/j.1541-0420.2006.00565.x 16918901

[7] Burt ML, Borchers DL, Jenkins KJ, and Marques TA. 2014 Using mark-recapture distance sampling methods on line-transect surveys. Methods in Ecology and Evolution 5: 1180–1191.

[8] Borchers DL, Zucchini W, and Fewster RM. 1998 Mark-recapture models for line transect surveys. Biometrics 54:1207–1220.

[9] Laake JL. 1999 Distance sampling with independent observers: reducing bias from heterogeneity by weakening the conditional independence assumption, in *Marine Mammal Survey and Assessment Methods* (eds. Garner GW, Amstrup SC, Laake JL, Manly BFJ, McDonald LL, and Robertson DG), Balkema, Rotterdam, pp. 137–148.

[10] Laake JL, and Borchers DL. 2004 Methods for incomplete detection at distance zero, in *Advanced Distance Sampling* (eds. Buckland ST, Anderson DR, Burnham KP, Laake JL, Borchers DL, and Thomas L), Oxford, Oxford University Press, pp. 108–189.

[11] Buckland ST, Rexstad EA, Marques TA, and Oedekoven CS. 2015 Distance sampling: methods and applications. New York, New York: Springer International Publishing.

[12] Becker EF, and Quang PX. 2009 A gamma-shaped detection function for line-transect surveys with mark-recapture and covariate data. Journal of Agricultural, Biological, and Environmental Statistics. 14:207–223.

[13] Strindberg S, Buckland ST, and Thomas L. 2004 Design of distance sampling surveys and geographic information systems, in *Advanced Distance Sampling* (eds. Buckland ST, Anderson DR, Burnham KP, Laake JL, Borchers DL, and Thomas L), Oxford, Oxford University Press, pp. 190–228.

[14] Quang PX, and Becker EF. 1999 Aerial survey sampling of contour transects using double count sampling technologies for aerial surveys, in *Marine Mammal Survey and Assessment Methods* eds. Garner GW, Amstrup SC, Laake JL, Manly BFJ, McDonald LL, and Robertson DG), Balkema, Rotterdam, pp. 87–97.

[15] Marques FFC, and Buckland ST. 2004 Covariate models for the detection function, in *Advanced Distance Sampling* (eds. Buckland ST, Anderson DR, Burnham KP, Laake JL, Borchers DL, and Thomas L), Oxford, Oxford University Press, pp. 31–47.

[16] Drummer TD, and McDonald LL 1987 Size bias in line transect sampling. Biometrics 43 13–21.

[17] Maindonald J, and Braun WJ. 2003 Data Analysis and Graphics using R. Cambridge University Press, Cambridge, UK. 525 pp.

[18] Burnham KP, Buckland ST, Laake JL, Borchers DL, Marques TA, Bishop JRB, et al 2004 Further topics in distance sampling, in *Advanced distance sampling* (eds. Buckland ST, Anderson DR, Burnham KP, Laake JL, Borchers DL, and Thomas L), Oxford, Oxford University Press, pp. 307–392.

[19] Marques FFC, and Buckland ST. 2003 Incorporating covariates into standard line transect analyses. Biometrics 59: 924–935. 10.1111/j.0006-341x.2003.00107.x 14969471

[20] Burnham KP, and Anderson DR. 1998 Model selection and multimodel inference: A practical information-theoretic approach 2^nd^ ed Springer-Verlag New York.

[21] Fewster RM, Buckland ST, Burnham KP, Borchers DL, Jupp PE, Laake JL. 2009 Estimating encounter rate variance in distance sampling. Biometrics 65:225–236. 10.1111/j.1541-0420.2008.01018.x 18363772

[22] Becker EF, and Christ AM. 2019 Rejection of Schmidt et al.’s estimators for bear population size. Ecology and Evolution 9:6157–6164. 10.1002/ece3.5134 31161027 PMC6540835

[23] Buckland ST, Laake JL, and Borchers DL. 2010 Double-observer line transect methods: levels of independence. Biometrics 66:169–177. 10.1111/j.1541-0420.2009.01239.x 19432793

[24] Laake JL, Borchers D, Thomas L, Miller D, and Bishop J. 2018. mrds: Mark-Recapture Distance Sampling. R package version 2.2.0 https://CRAN.R-project.org/package=mrds.

[25] Marshall L. 2020. dssd: Distance Sampling Survey Design. R package version 0.2.1.

[26] Strindberg S, and Buckland ST. 2004 Zigzag survey designs in line transect sampling. Journal of Agricultural, Biological, and Environmental Statistics. 9:443–461.

[27] Hedley SL, and Buckland ST. 2004 Spatial models for line transect sampling. Journal of Agricultural, Biological, and Environmental Statistics. 9, 181–199.

[28] Miller DL, Burt ML, Rexstad EA, and Thomas L. 2013 Spatial models for distance sampling recent developments and future directions. Methods in Ecology and Evolution, 4: 1001–1010.

[29] Nash JC, and Varadhan R. 2011 Unifying optimization algorithms to aid software systems users: optimx for R. Journal of Statistical Software 43(9): 1–14. URL http://www.jstatsoft.org/v43/i09/.

[30] Nash JC. 2014 On the best practice optimization methods in R. Journal of Statistical Software, 60(2): 1–14. URL http://www.jstatsoft.org/v60/i02/.

